# Toward an Understanding of Parental Views and Actions on Social Media Influencers Targeted at Adolescents: The Roles of Parents’ Social Media Use and Empowerment

**DOI:** 10.3389/fpsyg.2019.02664

**Published:** 2019-12-06

**Authors:** Meng-Hsien Lin, Akshaya Vijayalakshmi, Russell Laczniak

**Affiliations:** ^1^College of Business, California State University, Monterey Bay, Seaside, CA, United States; ^2^Marketing Area, Indian Institute of Management Ahmedabad, Ahmedabad, India; ^3^Department of Marketing, Iowa State University, Ames, IA, United States

**Keywords:** social media influencers, parents, adolescents, social media use, empowerment

## Abstract

Recent studies suggest that adolescents are spending significant amounts of time on social media. Brands are taking advantage of this fact and actively using social media to reach adolescent consumers, primarily *via* social media influencers. Adolescents consider the sponsored brand posts by social media influencers to be trustworthy and honest, thus reducing their critical evaluation of the ads. While several researchers have pointed to the critical role that parents play in their adolescents becoming digitally literate and empowered, there is little understanding of parental views and drivers of parental views on social media influencers and means by which they mediate their adolescents’ exposure to social media influencers. Our specific research questions are the following: (a) How does parents’ use of social media relate to their attitudes toward and mediation of social media influencers? (b) What is the role of psychological empowerment in enabling the relationship? Through a survey of approximately 200 mothers of adolescents (between the ages of 11 and 17 years), we examine how parents’ social media usage (active or passive) is related to their views toward social media influencers and mediation of social media influencers. We find that active (vs. passive) use of social media by parents led them to significantly (vs. not significantly) mediate social media influencers’ impact. Passive (vs. active) use of social media led to parents having a significant (vs. not significant) positive view of social media influencers. We explain this direct relationship by the level and kind of psychological empowerment (intrapersonal or interactional) that a parent experiences. Intrapersonal empowerment is related to self-efficacy, perceived competence, and desire for control, whereas interactional empowerment is related to an individual’s engagement in collective action and interactions with others. We find that active use of parental mediation increases intrapersonal empowerment resulting in parental mediation of social media influencers but has no effect on their positive or negative views on social media influencers. Moreover, passive use of social media results in interactional empowerment but has no significant impact on parental mediation but is related to positive views of social media influencers. Implications for regulators, practitioners, and parents are then discussed.

## Introduction

The presence of social media is everywhere. From a consumer perspective, its pervasiveness is enlightening and at the same time somewhat scary. Brands persuade consumers *via* Internet-based ads that are relatively obvious in their intent, such as banner ads posted on web pages, and in subtler ways, such as influencer posts on social media pages (Tutaj and van Reijmersdal, 2012). By many accounts, branded social media posts look and feel like regular, non-branded posts, making it difficult for consumers to differentiate them from each other. Furthermore, the interactive and immersive nature of Internet ads tends to increase the persuasiveness of a branded message (van Reijmersdal et al., 2010). Receivers of such messages require not just advanced cognitive skills and resources to defend themselves against persuasive attempts but also an understanding of how social media operates to recognize and effectively process branded social media posts. Thus, such messages would appear especially problematic for children. Children (including adolescents) are likely to have not yet developed the skills and abilities to determine the persuasive intent of branded social media messages. As a result, many parents may have negative views of branded social media messages (Nikken and Schols, 2015). On the other hand, the social media world does open up endless possibilities for learning among adolescents and children, and thus, parents may positively view this aspect of social media messaging. Given these potential discrepancies in the impact of social media brand messages, the main goal of this paper is to gain a better understanding of how parents view and manage branded social media posts that are targeted at adolescents.

Most of the research on parental views and mediation is limited to the context of traditional ads or slowly emerging in the context of native ads (e.g., Evans et al., 2013, 2018). Research with an aim to bridge this gap in understanding is particularly important when one considers that more than 70% of adolescents (12–15 years old) in the United States have a social media account (many of these social media users signed up for their accounts between the ages of 10 and 11; Ofcom, 2017). Moreover, it has been suggested that parents’ experiences in dealing with certain aspects of the Internet have led them to be less than effective in their own navigation of the Internet ad marketplace (Evans et al., 2013). Thus, parents’ views and mediation of branded social media posts targeted at their children are important in that they are likely to influence children’s responses to sponsored posts by social media influencers.

The broad objective of this research is to further our understanding of parents’ reactions to new emerging ads on the Internet. More specifically, through this paper, we attempt to understand parents’ views and mediation of branded posts by social media influencers. Through this, we will contribute to filling the research gap of understanding how social media is truly affecting consumers.

Prior research has substantiated that parental understanding and use of any media are likely to influence their views and mediation of it. We apply the constructs in the context of social media while also borrowing from Zimmerman’s (1995) theory of psychological empowerment to explain the above relationships better. Our specific research questions are the following: (a) How does parental use of social media relate to their attitudes toward and mediation of social media influencers? (b) What is the role of psychological empowerment in enabling the relationship? Psychological empowerment theory has been used to explain how active (vs. passive) social media use exerts its psychological effects in different types of empowerment (intrapersonal vs. interactional) (Leung, 2009; Li, 2016). Overall, both intrapersonal and interactional empowerment appears to provide more sense of control and availability of choice. However, the source of motivation and responses are fundamentally different from each other (Zimmerman, 1995; Leung, 2009; Li, 2016). We propose that such nuances are likely to lead to differences in parental attitudes and practices.

The method that we adopt to answer these questions is a survey of parents with children between the age of 11 and 17 years. The context is parental response to sponsored posts by social media influencers. The three main research questions we explore in our study are: (a) whether active, in comparison to passive, social media use has a significant relationship to mediation of social media influencers; (b) to understand how parents actively using social media are empowered by demonstrating higher self-efficacy, competency, and control (dimensions of intrapersonal empowerment). Combined, this would suggest that parents’ social media engagement is likely to drive their level of comfort (understanding) and confidence (through empowerment) with the way social media operates and therefore helps them engage with their adolescents about social media marketing tactics. Hence, in the third objective, we examine (c) how the passive use of social media is likely to result in parents holding a positive view of social media influencers but demonstrate a lack of involvement in mediation. And is this the case because passive-use parents tend to rely on their community (interactional empowerment) to guide their views on various issues. Overall, we expect to find that there are benefits from using social media actively and become more empowered as a parent, whereas there are pitfalls of relying on the community (arising from passive use of social media) to handle adolescent’s media-related decisions.

The research is timely because social media influencers are growing in numbers, and there is little understanding about this phenomenon on consumer actions and reactions. By grounding our research in psychological theory, we provide significant implications for policy makers and parents. Finally, we contribute to consumer socialization literature by considering the consumer behavioral construct—active or passive social media use—which richly captures consumer engagement on the Internet.

## Background

The concept of influencers is not new. Its origin can be traced to traditional media and the presence of opinion leaders who for the purpose of persuasion lead discussions on specific topics related to their expertise (Zhao et al., 2018). Extending this definition to social media, users who started as a regular person or are famous in a field and who have accumulated a large number of followers on one or more of the online media platforms (e.g., Instagram, TikTok, YouTube) and often persuade followers through their authentic messages are considered as social media influencers (Lou and Yuan, 2019). Social media influencers often have gained popularity on these media for their expertise or interest in some areas, such as food, fashion, or lifestyle (Lou and Yuan, 2019). The influencers can have followers from anywhere in the tens of thousands (micro-influencers) to the millions (celebrity influencers).

Influencer marketing, a form of social media marketing, occurs when brands use social media influencers to drive their brand awareness, conduct product placements, and endorse products on their personal social media pages to increase purchase intentions of that brand among their consumers or social media influencers’ followers (Lou and Yuan, 2019). Influencer marketing could take the forms of status message, brand integration, or sweepstakes (van Dam and van Reijmersdal, 2019). The social media influencers, in return for endorsing the brand, get paid or are offered free products by the firm. Firms are widely adopting tactics of brand promotions by influencers because of its effectiveness. In 2018, there were 3.7 million brand-sponsored posts on Instagram, and this is suggested to rise to 6.12 million by 2020 (Mediakix, 2019); whereas the global marketing spending on influencers is likely to rise from $4.17 billion in 2018 to $8.08 billion in 2020 (Influencer DB, 2018), indicating that this is a growing trend in social media marketing. Almost all marketers are beginning to use social media influencers, including B2B brands because they yield return on investments, which are significantly higher than that of traditional media (Ahmad, 2018). Furthermore, many marketers who have used social media influencers to spread their brand message tend to believe in its effectiveness (Folkvord et al., 2019). Surveys also suggest that users tend to purchase more based on the influencer’s recommendations (Lou and Yuan, 2019).

Identifying such opinion leaders or influencers to spread the word helps firms effectively use their limited marketing resources (Zhao et al., 2018). The social media influencers tend to be cheaper than the use of celebrities (Lou and Yuan, 2019). Not only is influencer marketing more cost-effective than hiring celebrities, most of the time, it is also significantly cheaper than mass media advertisements. Moreover, the brand message can be easily personalized and varied from one influencer to the other in comparison to a standardized message on TV or print. This allows brands to adapt to different target markets and demographic profiles, and even to different geographic locations and local cultures, resulting in a win–win situation for brands and firms. In the era of consumers opting out of ads and using AdBlock on websites, the most significant advantage for brands using influencer marketing is the fact that users choose to follow the social media influencers and hence are more open to engaging with the influencer’s posts. Moreover, many users are not aware that the influencers receive something in return from the firm for their endorsement, which makes the user believe a sponsored brand message as a personal recommendation (Ofcom, 2017).

### Social Media Influencers and the Process of Influencing

“Influencers” are so called because they impact learning and, thus, cognitions and behaviors *via* “modeling, reinforcement, and social interaction” (Folkvord et al., 2019; p. 79). Social media, such as Facebook and Instagram, encourage users to engage *via* liking and/or commenting on people’s posts. Such interactions tend to build social bonds among the users and increase their attachment and emotional belongingness to the community (Zeng et al., 2017). Users tend to do this for not just their family/friends’ posts but also to unrelated influencers’ posts. That is, the influencing happens when a child or an adult user watches/reads, likes, comments/interacts with the social media influencer on a sponsored brand post uploaded by that individual (van Dam and van Reijmersdal, 2019), utilizing their social capital. Through such interactions with influencers, social bonds are created, and users become more open to social influencer’s user-relevant product endorsements (Zeng et al., 2017). Adolescents often build strong relationships with the influencers, and one “with whom you have strong relationships are usually not expected to have ulterior motives” (van Dam and van Reijmersdal, 2019; p. 2). Social media influencers, therefore, can be considered a social agent influencing an adolescent’s consumer socialization process.

The product endorsements or brand messages posted by social media influencers, in contrast to typical advertisements, appear like personal status messages, thus, seamlessly promoting the brand as if it is a personal recommendation from one’s friend or family member. Such personal recommendations are considered differently from advertisements by users, and they tend to have a more positive brand effect on the users (De Jans et al., 2018). Moreover, many times, because of the influencers’ initial roots as regular users, followers tend to relate to them easily and find them credible (Swant, 2016) to the extent that many users consider social media influencers as their peers (van Dam and van Reijmersdal, 2019). In addition, peer influence has been shown to have a significant impact on children’s desire for brands, especially in online social settings (Rozendaal et al., 2013). Furthermore, influencers tend to possess and provide first-hand information about a brand, thus acting as firm insiders (Zhao et al., 2018) and opinion leaders. For example, Colgate launched its SlimSoft Charcoal Toothbrush using 200 social media influencers targeting approximately 24 million customers on social media. These influencers received different black items (e.g., a black mug, chocolate inside a black egg) over 3 days. They posted these items along with the hashtag WhatTheBlack to build up curiosity among their followers and subsequently unveiling the toothbrush on the last day (Sinha, 2016). This kind of exclusive access to brand information that influencers possess increases the hype and trust of the consumers toward the influencers. This extends then to the brand and product endorsed because the users believe that it is “tested and proven” by someone they know. The messages posted by the influencers also tend to be organic and have a personal voice or personality (rather than a standard and commercial message from the brand) (Lou and Yuan, 2019). Furthermore, it is suggested that this type of engagement between consumers and brand *via* influencers increases feelings of familiarity and liking for a brand (Coates et al., 2019).

### Social Media Influencers and Adolescents

Social media use has become especially habitual among adolescents, which also means that they are exposed to many native advertisements on a daily basis (Lou and Yuan, 2019). Influencer posts are a form of native advertising because the intent of commercial persuasion is masked by personal message curated by the social media influencer (van Dam and van Reijmersdal, 2019). Even institutions engaged in crafting media regulations are beginning to acknowledge the persuasive impact of subtle messaging by influencers. For instance, United Kingdom’s advertising regulatory board has suggested that any user with 30,000 followers be classified as a celebrity, thus, requiring them to follow advertising rules related to product endorsements (Porter, 2019).

Children are suggested to find influencers more relatable and credible, often aspiring to achieve their lifestyles (De Jans et al., 2018). These influencer messages are also considered by children to be more trustworthy and honest than other commercial messages (Paek et al., 2011; De Jans et al., 2018). In fact, Lou and Yuan (2019) find that purchase intention was primarily driven by the trustworthiness of the influencer. They reported that trust in branded posts and educational value, rather than the influencer’s level of attractiveness or entertainment value of the message, was vital in influencing a user’s purchase decisions, thus prompting many brands to reach children *via* social media influencers.

Children between the ages of 9 and 11 years increased their intake of calories and unhealthy snacks when exposed to social media influencers who endorsed the same (Coates et al., 2019). Similarly, Folkvord et al. (2019) find that children most often recalled high-energy dense snacks, such as offerings from KFC or McDonald’s, which appeared in vlogs (videos posted by influencers on YouTube). The subtle and embedded nature of the brand recommendation in these vlogs is likely to reduce elaboration and counterarguments and lower defenses against commercial messaging (Cornish, 2014). This could be expected to be even more so for children because they are still developing their abilities to process persuasion messages (Brucks et al., 1988). Because brand promotion by influencers often utilizes arousing or emotional message appeals, children’s cognitive resources might be used to process the entertaining aspect of these promotions rather than activating persuasive knowledge, which is critical for analyzing the brand message (Folkvord et al., 2019). Moreover, it is suggested that the affective rather than the cognitive areas of the adolescents’ brains are more (vs. less) reactive, thus making them even more susceptible to sponsored social media posts (Defoe et al., 2015). When adolescents are unable to identify persuasive intent in brand messages, they are more likely to be susceptible to its effects (Cornish, 2014), hence, showing higher purchase intent. Furthermore, van Dam and van Reijmersdal (2019) find that while children may understand how influencers worked, they lack a critical attitude and were more accepting of the practice of influencers posting sponsored messages. This is likely because the motivation to critically analyze the post by someone whom the adolescent user is voluntarily following on social media is low as compared to the motivation to critically process a non-native ad (e.g., banner ad), which is not chosen by the user (Folkvord et al., 2019).

In short, van Reijmersdal et al. (2012) argue that the new ways of advertising lead to information processing, which is different from processing of traditional ads. Therefore, we might have to reconsider how to prepare an adolescent better to handle the new forms of native advertisements. In that regard, we consider parents’ own use of social media as an initial step toward understanding social media marketing tactics and better preparing an adolescent to handle the incoming persuasive messages.

### Social Media Influencers and Parents of Adolescents

Even though there are policy recommendations in the United States, United Kingdom, and Europe, recommending social media influencers to identify advertisements and differentiate them from regular posts, there are no mechanisms to enforce this, and violators of this recommendation are rarely held responsible. Hence, it is generally assumed that the responsibility of managing these branded posts rests with the parents. Parents are considered as “gatekeepers of children’s exposure to online persuasive messages” (Cornish, 2014; p. 438), requiring them to reduce their children’s contact to these messages through screening, discussions, and filtering. In addition to acting as gatekeepers, we propose that parents also play a significant role in preparing and empowering their adolescent to handle various types of Internet ads.

Over the past years, multiple studies conducted across countries have concluded that parents play a critical role in consumer socialization, especially increasing children’s advertising literacy (Nelson et al., 2017). Research conducted by Livingstone (2008) and Livingstone et al. (2011) shows that parents have an impact on adolescents’ positive or negative engagements on the Internet. For example, the authors suggest that co-surfing or active media-related discussions by parents is likely to improve a child’s Internet skills. However, for parents to effectively play their role as a gatekeeper or assist in their adolescent’s consumer socialization process, they have to demonstrate an understanding of social influencer’s posts. This could be a stumbling block as researchers have found that, for instance, parents demonstrated a poor understanding of advergames (another type of native Internet advertisement) even after being provided with a definition for it (Evans et al., 2013). Poor understanding of Internet ads in parents results in ineffective ad-related discussions with their children (Evans et al., 2018). Moreover, Cornish (2014) finds that parents tend to dismiss the effects of online advertising as compared to the effects of their child’s safety on the Internet, such as online predators and cyberbullying. The author suggests that this indifferent attitude toward Internet ads is likely to be carried over to their children as well, thus making both parents and children more susceptible to online ads (Cornish, 2014). When parents themselves are vulnerable to Internet ads or tend to discount their effects, they are less likely to mediate their children’s ad exposure (Pettigrew et al., 2013). Thus, we expect that parents’ understanding of ads and attitude toward social media influencers are critical to predicting the actions (i.e., parental mediation) taken to manage the impact of social media influencers on their adolescent.

Parental mediation involves the means and methods by which parents manage their children’s media use (Shin and Kang, 2016). It rests on the premise that children are affected by the use and exposure to social media, and parents can play a significant role in mitigating the adverse effects of such exposure (Shin, 2010). Parental mediation involving discussions about social influencer ads, co-surfing social media, monitoring child’s social media usage, and having rules for social media use are likely to positively influence children’s understanding of social media ads (American Academy of Pediatrics, 2016; Evans et al., 2018). For instance, active co-surfing of social media with children will provide parents with information about their child’s activities and opportunities to discuss the role of social media influencers in the advertising world (Evans, 2014). Setting rules for media consumption tends to lower screen time in children (Yang et al., 2017). Evans et al. (2018) suggest and find that the use of one kind of parental mediation will increase the chances of using other mediation techniques. Hence, in this study, we focus on overall parental mediation, including the use of multiple means such as rule making or co-surfing rather than considering each method individually.

**FIGURE 1:**
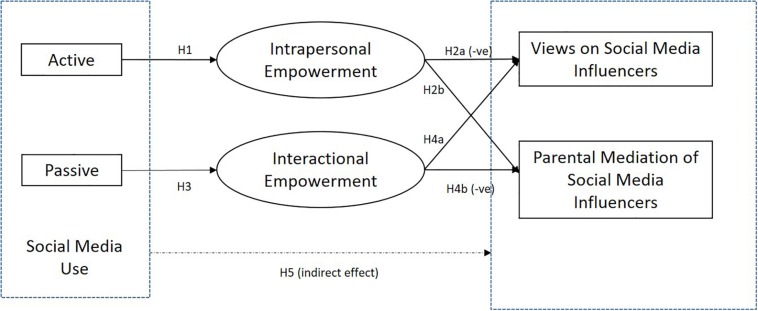
Proposed model.

Cornish (2014) argued that despite acknowledging the significant role that parents play, we know little about parents’ roles in managing Internet ads. This goes for parental views and mediation of social influencer posts as well. Therefore, in this paper, we focus on understanding parents’ perceptions of social media influencers by (a) examining how parents engage with social media in general, capturing the nature and patterns of their social media activities by considering passive vs. active use of social media, (b) relating such social media use with parental attitudes toward and how parents manage the impact of social media influencers on their adolescent, and (c) investigating the mediating role of psychological empowerment developed through social media use to explain the mediation methods taken to manage social media influencers. We respond (Figure 1) to the call for the special issue of Frontiers Psychology, The Role of Social Media Influencers in the Lives of Children and Adolescents, which urges researchers to investigate “parental attitudes and mediation styles with regard to social media influencers.”

## The Present Study

### Active and Passive Social Media Use

People use social media for various purposes—for social networking, learning, and entertainment interests and to express political views (Yu, 2016), among many other reasons. Many parents, especially, suggest that social media networks provide them useful information (Duggan et al., 2015). In general, individuals’ activities on social media can be broadly divided into two types—viewing and posting. Posting activities include sharing opinions or photos or videos, seeking responses to questions, sharing personal information and knowledge, or responding to other user’s posts on social media (Pagani et al., 2011). Posting can be considered as “active use” of social media, while mere reading or watching can be considered as “passive use” of social media (Yu, 2016). For active users, social media provides an outlet to express their identity, beliefs, and emotions (Escobar-Viera et al., 2018), fulfilling requirements beyond utilitarian needs. Passive use of social media is limited to watching videos and reading posts by others, and the engagement occurs only at the surface with very little or no interaction with others or social media content online.

However, passive use does not necessarily mean that a user is spending less time on social media; hence, type of social media use should be seen differently from time spent on social media. In fact, how people use social media should also be differentiated from their Internet literacy or skills. While Internet skills focus on one’s ability to access online facilities and functions to use them effectively (Livingstone et al., 2005), active use is related mainly to the individual’s level of activity in terms of posting and commenting on social media. The two are not necessarily always capturing the same thing. That is, people who have more knowledge and are fluent with using the Internet (high Internet skills) may not necessarily post or comment a lot (active social media use). Moreover, research finds that high Internet skills do not automatically indicate a higher understanding of the subtleties of Internet advertising (Cornish, 2014). Hence, capturing active (vs. passive) use of social media may be a more effective way to explain such scenarios as we illustrate below.

For parents to mediate the impact of social media influencers, they will first have to demonstrate awareness about ad targeting *via* social media posts and show understanding of how individuals might be affected by it (Pettigrew et al., 2013). That is, parents need to be able to identify a native ad as well as demonstrate skepticism toward it in order to mediate children’s exposure to social media influencers. Such understanding is likely to come from increased use of social media. On the marketing front, researchers found that increased social media use leads to higher brand engagement or increased expression of political beliefs (Gil de Zúñiga et al., 2012). Parents’ media use influences their involvement in and guidance of their children’s media use (Nikken and Schols, 2015). For instance, the same authors find that parents’ media use has a significant effect on the time a child spent on TV, gaming devices, computers, and touch screen devices. Studies in the context of children’s vaccination decisions suggest that parents who had the knowledge and were information-seeking were more confident about their vaccination decisions, whereas parents who were information avoiders tended to make less informed medical decisions (Fadda et al., 2016). Furthermore, parents with a critical attitude toward social media were more likely to mediate their children’s social media use (Daneels and Vanwynsberghe, 2017). Based on these findings, we expect active (vs. passive) parent users of social media to gain knowledge and competence from their use of the media, leading to more negative (vs. positive) views of social media influencers, and, at the same time, result in increased (vs. reduced) parental mediation of social media influencers. We explain the process and hypotheses below.

### Psychological Empowerment as a Mediator Between Social Media Usage and Parental Views and Mediation

“Empowerment is the process by which people increase control over their lives and health” (Fadda et al., 2017; p. 100). Psychological empowerment is defined in this context as the intrinsic motivation in parents to understand the role and impact of social media influencers on their adolescent’s social media use. The role of psychological empowerment in parenting practices has traditionally been limited to the study of parents with children in special education and needs (Murray et al., 2013). More recently, there are some studies on the impact of the empowering role of social media blogging for new mothers (McDaniel et al., 2012). In another study, the authors focused on how social media provides a source of information and social support for new mothers (Madge and O’Connor, 2006), which is positively correlated with feelings of connectedness, thus predicting the sense of well-being. Hsieh et al. (2018) find that social media use enhances psychological empowerment resulting in customer value co-creation in online brand communities. Other online contexts include employee’s use of social media to showcase their commitment to the organization (Gümüş et al., 2016) as a form of psychological empowerment.

Psychological empowerment has several dimensions, including intrapersonal, interactional, and behavioral (Zimmerman, 1995). Intrapersonal empowerment explains how individuals think and feel about themselves and is understood through an individual’s level of self-efficacy, perceived control, and perceived competence (Zimmerman, 1995, 2000; Li, 2016). This dimension measures an individual’s beliefs in their competency to take proactive steps to affect their environment (Fadda et al., 2017). Self-efficacy refers to the belief that an individual has about their capability to perform certain behaviors to complete the tasks necessary to reach a goal (Bandura, 1977), whereas perceived control refers to the power to change or influence situations in the domain of interest. Specifically, in our case, it is the perception of control a parent has over mitigating the likely impact of social media influencers on their adolescent. Finally, perceived competence refers to an individual’s belief in the skills and abilities one possesses to have control over the circumstances and thus make the desired change (Menon, 2001).

A study found that user-generated content online, an active form of social media use, enhanced psychological empowerment, and in turn, increased civic engagement offline (Leung, 2009). Similarly, parents who feel more competent tend to be more knowledgeable and show more confidence in decisions for their children (Fadda et al., 2017). Active (vs. passive) use of social media may create feelings of efficacy (that one’s action can have an impact), beliefs in one’s competence to understand and utilize social media effectively (Yu, 2016). Moreover, Vijayalakshmi et al. (2018) found that parents with high parental efficacy are likely to believe that it is their responsibility to manage their children’s media use. These beliefs are expected, along with parents’ own encounters with social media influencers, to result in a more critical attitude toward social media influencers. Furthermore, active use of social media is likely to increase intrapersonal empowerment in parents, increasing their likelihood to mediate social media influencers’ impact. Therefore, we hypothesize that:

H1: Active social media use is likely to be positively related to intrapersonal empowerment.

H2: Intrapersonal empowerment is likely to be positively related to (a) negative views on social media influencers and (b) parental mediation.

On the other hand, passive use of social media lacks this feedback loop, which is critical in developing and strengthening qualities related to self-efficacy and perception of control. This positive reinforcement is crucial and can be gained only through active involvement in discussions and interactions with acquaintances and non-acquaintances alike on social media. Hence, passive use is not expected to result in intrapersonal empowerment.

Interactional empowerment, the other dimension of psychological empowerment, is the belief in actions and performance that can be achieved through collective efforts (Li, 2016). An interactionally empowered individual may focus on understanding the opinions and experiences of one’s peers (Fadda et al., 2017). Interactional empowerment, as the term suggests, is parents’ access to and collaboration with networks or community, which can help them in bringing about the desired change (Kim and Bryan, 2017). It can be conceptualized as one’s reliance on collective action and interpersonal relationships (Li, 2016). Li (2016) has shown that interactional empowerment is related to passive social media use. Passive use of social media involves reading and consuming information on social media, where activities are limited to those not related to the creation of content, and interactions are more one-way in nature (i.e., listening to people’s views and opinions). In such cases, beliefs of change and actions are formed but mainly through reliance on “others” or when parents “act together” to achieve these goals. It is suggested that intrapersonal and interactional empowerment tends to operate in opposite ways (Fadda et al., 2017). Parents who tend to depend on external support felt less autonomous to make decisions, including for critical decisions such as vaccinating their child (Fadda et al., 2017). In the context of perceptions and mediating social media influencers, external support that could have an influence include media regulation associations, schools, the government, parent groups, or even the social media firms themselves. Such belief places the agency of change on a larger entity than the self. Hence, parents who gain interactional empowerment from passive use are not expected to have a critical view of social media influencers. Moreover, they are unlikely to be directly engaged in parental mediation of social influencers because agency of change is placed in the broader environment or entities, such as government agencies, teachers, or businesses who manage the social media sites (e.g., YouTube, Instagram, etc.) for curbing any negative influences (Evans et al., 2018; Vijayalakshmi et al., 2018). As a result, we expect:

H3: Passive social media use is likely to be positively related to interactional empowerment.

H4: Interactional empowerment is likely (a) to be positively related to positive views on social media influencers and (b) unrelated to parental mediation.

Based on the above research, overall, we expect parents who actively use social media to be intrapersonally empowered and, in turn, more likely to have critical views and mediate their adolescent’s exposure to social influencer’s branded posts.

H5: Intrapersonal (vs. interactional) empowerment is likely to mediate the relationship between active (vs. passive) use of social media and (a) negative (vs. positive) view of social media influencers and (b) parental mediation.

### Study Expectations

The current study is based on a cross-sectional survey for the purpose of better understanding the influence of parental social media use on their (a) views on social media influencers and (b) mediation of the impact of social media influencers on their adolescent. To further explain the underlying mechanisms of these relationships, we examine the possible mediating role of psychological empowerment developed from social media use. The model is tested using survey data collected from parents of adolescents between the ages of 11 and 17 years. Based on prior research findings, we expect that active social media users are likely to have higher intrapersonal empowerment (H1), which is then related to a critical view of social media (H2a) and increased parental mediation (H2b), whereas passive social media users are likely to demonstrate higher interactional empowerment (H3), which is related to a more positive view of social media (H4a) and reduced mediation (H4b). Overall, we expect intrapersonal (vs. interactional) empowerment to mediate the relationship between active (vs. passive) use of social media and (H5a) more (vs. less) skeptical view of social media influencers and also (H5b) more (vs. less) likely attempts to mediate the impact of social media influencers.

## Materials and Methods

### Participants and Sampling Procedures

The sample for this survey was recruited through the use of Qualtrics panel. Qualtrics provides Internet-based data collection services, such as access to consumer panels (Kees et al., 2017). Qualtrics invited participants to engage in the survey by posting the survey link (along with the participant criteria) on their message board and also by emailing their panel members. A total of 425 parents in the United States Qualtrics panel were emailed the survey. Participants were next screened with the qualifications of being at least over the age of 18 (not minors), female, and had at least one child between the ages of 11 and 17, which resulted in 268 participants who qualified for and started the survey. We focused on mothers because previous research has found that mothers are the primary caretakers, socializers, and more likely to be involved in mediating their adolescent’s advertising (Kowalczyk and Royne, 2016). Furthermore, mothers are more likely than fathers to use social media platforms, such as Facebook, Pinterest, and Instagram. Mothers (vs. fathers) are also likely to log in to Facebook more (vs. fewer) times a day as well as engage more (vs. less) on Facebook (Duggan et al., 2015).

The survey took approximately 15 min to complete. Two attention check questions were used to ensure that participants were actively engaged in the completion of the survey. Furthermore, participants who took less than 6 min, one-half the median time, to complete the survey were removed from the final sample out of concerns that they were not responding thoughtfully. This resulted in a total of 208 participants whose data could be considered for further analyses. We conducted further data checks and removed participants who either did not report the grade level for their children or reported fewer people in the household than the number of children and participant combined (indicating a lack of consistency in responses) and removed one participant who was 73 years old (all others were under 60). As a result, the final sample consisted of 182 mothers of adolescents between the ages of 11 and 17 years. If the parents had two or more adolescents between the ages of 11 and 17 years, we asked them to consider the oldest child when answering the questions.

The average age for the mothers who participated in the study was 40 years old, 63.2% of the participants had an annual household income of 75,000 or less, 50% of the respondents worked a full-time job, whereas 36% of the mothers did not work for wages. Fifty-six percent of the respondents held at least a college degree. Twenty-seven percent of the mothers had only one child, 86% of them were white, and 75% of the mothers were married at the time they took the survey. The majority (87%) of the parents reported as having adolescents who were referred to in the survey as currently attending 7th to 11th grade.

Out of the 182 participants, 98% of the mothers owned a smartphone, and all participants had access to some form of social media. Ninety percent of the participants used social media (such as Facebook, Twitter, Instagram, LinkedIn) regularly, whereas others used social media occasionally. Adolescents spent significantly more time on the Internet on the weekends (*M* = 4.60 h) compared to weekdays (*M* = 3.60 h) [*t* (181) = 5.38; *p* < 0.001]. In Table 1, we provide the complete sociodemographic details of the participants and their adolescents.

**TABLE 1:** Demographic characteristics of the participants.

	***N* = 182**
**Parents’ characteristics**	***M* (*SD*)**

Age (years)	40.27 (7.21)

**Parents’ education**	***n* (%)**

Basic or secondary studies	83 (45.6)
Higher education	99 (54.4)
(associate, bachelor’s, master’s, or doctoral degree)	

**Parents’ marital status**	***n* (%)**

Married	138 (75)
Widowed/divorced/separated	25 (13.6)
Never married	19 (10.3)

**Number of children**	***n* (%)**

One	50 (27.2)
Two or more	132 (72.8)

**Household income**	***n* (%)**

<$74,999	115 (63.2)
>$74,999	67 (36.8)

**Employment status**	***n* (%)**

Full-time	92 (50)
Part-time	23 (12.5)
No	67 (36.4)

**Adolescent’s characteristics**		
**Child grade**	***n* (%)**

4th grade	1 (0.5)
5th	3 (1.6)
6th	15 (8.2)
7th	24 (13)
8th	20 (10.9)
9th	43 (23.4)
10th	38 (20.7)
11th	33 (17.9)
12th	5 (2.7)

**Hours spent on social media by child**	***M* (*SD*)**

On weekdays	3.60 (2.57)
On weekends	4.40 (2.60)

### Measures

#### Parental Views on Social Media Influencers

The survey included questions on the parent’s beliefs and perceptions about social media influencers. We included a short description and an example of an Instagram post of a social media influencer (see Supplementary Material for details). We then asked the parent to respond to statements regarding their perceptions of social media influencers. This part of the survey included a total of 10 statements, including four positive and six negative thought statements adapted from Evans et al. (2013). The authors used them for measuring parental attitudes toward advergames. Participants were asked to rate the items on a five-point Likert scale ranging from (1) strongly agree to (5) strongly disagree. Sample questions for negative thoughts (α = 0.87) included “Social media influencers advertising brands take undue advantage of children” and positive thoughts (α = 0.75) “There is nothing wrong with social media influencers sponsored by brands.”

#### Parental Mediation Scale

Parental mediation regarding the methods used to manage social media influencers (α = 0.87) were adapted from a scale developed by Nimrod et al. (2019) specifically for Internet mediation. Items were based on both prior mediation scales and recommendations by researchers on the need to include mediation techniques, such as monitoring for Internet mediation. Parents were instructed to identify how often they engaged in the following activities with their adolescent, including eight statements that captured parents’ use of restrictive, instructive, supervision, and co-use mediation to manage children’s interactive media use. Sample questions such as the following were asked, “Specify when and for how long your children can use social media” or “Stay in the same room and keep an eye on the screen when the child uses social media.” Parents rated the items on a five-point scale indicating the frequency of participating in such actions from (1) never to (5) always. For the subsequent analysis, we aggregated and averaged the eight items similar to Evans et al. (2018). All of the items were significantly correlated with each other (*p* < 0.05), suggesting that parents’ use of one kind of mediation led to use of the others as well (Evans et al., 2018).

#### Social Media Use

A user is considered to be active on social media if they create content, contribute to group discussions, and post comments on the pages, whereas passive users merely consume (*via* reading or watching) the content others produce (Pagani and Mirabello, 2011). Based on this definition, to measure the participant’s use of social media, passively or actively, we adapted scales from Leung (2009) and Li (2016). Participants were asked to rate their level of social media use on a five-point scale from (1) never to (5) always for eight different items capturing their activity on social media. Three of the actions related to passive use (α = 0.84), such as “I read online discussions” or “I watch videos or pictures posted on social media sites.” The five other actions were considered as active use (α = 0.86), such as “I post content on my own social media page” or “I share content on social media sites with my connections.”

#### Psychological Empowerment

This measure was also adapted from Leung (2009) and Li (2016). It had three indicators for intrapersonal (self-efficacy, perceived competence, and control) and two indicators for interactional (collective action and interpersonal relationships) empowerment. They were evaluated using a five-point Likert scale, with participants rating their level of agreement with the statements ranging from (1)strongly disagree to (5) strongly agree. A sample item for self-efficacy (α = 0.91) included “I am confident that I could deal efficiently with unexpected events.” For perceived competence (α = 0.77), the items included “I am often a leader in groups,” and for control (α = 0.69), “I enjoy making my own decisions.” Sample items for collective action (α = 0.67) included “Power in the online community lies in the relationships between people” and for interpersonal relationships (α = 0.70) “Only by working together can people exert influence in the online community.” The reliability scores, while appearing to be slightly low, are in line with the alphas reported by Leung (2009) and Li (2016) in their studies, and the items are considered adequate measures of the given constructs.

#### Parental Internet Skills and Other Background Variables

To capture the level of Internet skills (Shin, 2010), 18 items (α = 0.91) were used to measure an individual’s ability to navigate the online sphere. Participants were asked to rate on a five-point scale from (1) poor to (5) excellent, activities such as “Write a blog or online diary,” “Use instant messaging,” and “Watch video clips.” Additionally, the demographic details for the mother and child were also captured. This included the mother’s age, marital status, household income, education level, work status, ethnicity, number of children, adolescent’s grade level, and adolescent’s use of social media.

## Results

Data analysis was conducted using Statistical Package for the Social Sciences (IBM^®^ SPSS^®^, version 26.0; IBM SPSS, Chicago, IL, United States) and AMOS 26 (IBM^®^ SPSS^®^ AMOS^TM^ Version 26.0; IBM Corporation, Meadville, PA, United States). Reliability tests of the constructs were conducted using SPSS (presented in Table 2). Additionally, Pearson correlations (also calculated *via* SPSS, see Table 3) were computed to identify if there were any bivariate associations between the sociodemographic variables and other critical independent or dependent variables of the model. The final model was tested using AMOS.

**TABLE 2:** Descriptive statistics of the independent and dependent variables.

	**Reliability Alpha**	
	**(number of items)**	**Mean (*SD*)**
**Social media use**		
Passive use of social media	0.84 (3)	3.13 (1.10)
Active use of social media	0.86 (5)	2.83 (1.07)
**Intrapersonal empowerment**		
Self-efficacy	0.91 (5)	3.47 (0.96)
Perceived competence	0.77 (4)	3.13 (1.03)
Control	0.69 (3)	3.77 (0.87)
**Interactional empowerment**		
Collective action	0.67 (3)	3.46 (0.80)
Interpersonal relationships	0.70 (3)	3.67 (0.74)
**Parental response**		
Positive views on social media influencers	0.75 (3)	3.01 (0.85)
Negative views on social media influencers	0.87 (6)	3.72 (0.78)
Parental mediation	0.87 (8)	2.97 (0.93)

**TABLE 3:** Correlation between the dependent and independent variables.

	**1**	**2**	**3**	**4**	**5**	**6**	**7**	**8**	**9**	**10**
1. PUSE	1									
2. AUSE	0.67^∗∗^	1								
3. SE	0.27^∗∗^	0.32^∗∗^	1							
4. PerComp	0.22^∗∗^	0.27^∗∗^	0.54^∗∗^	1						
5. Control	0.24^∗∗^	0.21^∗∗^	0.39^∗∗^	0.36^∗∗^	1					
6. CollAct	0.34^∗∗^	0.34^∗∗^	0.29^∗∗^	0.26^∗∗^	0.32^∗∗^	1				
7. IntPerRe	0.33^∗∗^	0.29^∗∗^	0.25^∗∗^	0.19^∗∗^	0.21^∗∗^	0.64^∗∗^	1			
8. PosInf	0.26^∗∗^	0.27^∗∗^	0.14	0.02	0.00	0.37^∗∗^	0.34^∗∗^	1		
9. NegInf	0.03	–0.02	0.05	0.04	0.16^∗^	–0.02	0.07	–0.43^∗∗^	1	
10. ParMed	0.26^∗∗^	0.30^∗∗^	0.28^∗∗^	0.27^∗∗^	0.08	0.11	0.15^∗^	0.06	0.03	1

*PUSE, passive use of social media; AUSE, active use of social media; SE, self-efficacy; PerComp, perceived competence; Control, perceived control; CollAct, collective action; IntPerRe, interpersonal relations; PosInf, positive influence; NegInf, negative influence; ParMed, parental mediation. ^∗^*p* < 0.05 and ^∗∗^*p* < 0.01.*

### Correlation Between the Variables

The results of correlations (Table 3) showed a positive and significant association (*p* < 0.05) between active use of social media and the following variables: passive use of social media (0.67), the indicators of intrapersonal empowerment (self-efficacy: 0.32; perceived competence: 0.27; control: 0.21) and interactional empowerment (collective action: 0.34; interpersonal relations: 0.29), positive thoughts about social media influencers (0.27) and parental mediation (0.30). Similarly, passive use of social media positively and significantly correlated to the following variables: the indicators of intrapersonal empowerment (self-efficacy: 0.27; perceived competence: 0.22; control: 0.24) and interactional empowerment (collective action: 0.34; interpersonal relations: 0.34), positive thoughts about social media influencers (0.26), and parental mediation (0.26). The indicators of both interactional and intrapersonal empowerment latent variables were also correlated to each other (Table 3). Positive thoughts about social media influencers were significantly and inversely correlated to negative thoughts on social media influencers (−0.43). However, neither of the above thought variables was significantly correlated to parental mediation. Parental mediation was positively correlated to some of the indicators of intrapersonal empowerment (self-efficacy: 0.28; perceived competence: 0.27) and interactional empowerment (interpersonal relations: 0.15).

Among the demographic variables, we find that a child’s grade level in school had a significant negative relationship with parental mediation (−0.21), the dependent variable, suggesting that parents are less likely to mediate as their adolescent grows older. None of the other demographic variables, including parental income, marital status, and ethnicity had any significant impact on the independent or the dependent variables. Parental Internet skills also had a significant and positive relationship with the independent variables: active use of social media (0.36), passive use of social media (0.42), intrapersonal empowerment (self-efficacy: 0.40; perceived competence: 0.28; control: 0.28), and interactional empowerment (collective action: 0.22; interpersonal relations: 0.20). Hence, the child’s grade level and parental Internet skills were included as covariates in the model.

### Model Specifications

To examine the impact of parents’ social media use on parenting related to social media influencers, a confirmatory factor analysis (CFA) was conducted using AMOS. The final sample consisted of 182 mothers of adolescents between the ages of 11 and 17 years. We also conducted a bootstrap resampling procedure with 2000 samples and 95% bias-corrected confidence interval. Based on this, we were able to estimate the direct and indirect effects; results are provided in Tables 4, 5. As per our proposed model, we expect passive/active social media use to be significantly related to positive/negative thoughts about social media influencers and levels of parental mediation and the relationship to be mediated by psychological empowerment. More specifically, we expect that (a) active social media use would directly influence intrapersonal empowerment, (b) passive social media use would influence interactional empowerment, (c) intrapersonal empowerment would increase negative thoughts about social media influencers and parental mediation, whereas (d) interactional empowerment would increase positive views on social media influencers and reduce parental mediation. And finally, we expect based on prior research (e) positive (vs. negative) thoughts to reduce (vs. increase) parental mediation.

**TABLE 4:** Direct effects of independent variables on dependent variables.

**Dependent variables:**	**Intrapersonal empowerment**	**Interactional empowerment**	**Positive thoughts**	**Negative thoughts**	**Parental mediation**
**Independent variables:**					
**Social media use**					
Active use	0.20 (0.06, 0.30)^∗^	0.10 (−0.04, 0.24)			
Passive use		0.16 (0.05, 0.27)^∗^			
**Intrapersonal empowerment**				−0.09 (−0.42, 0.23)	0.68 (0.05, 1.22)^∗^
Self-efficacy	1.00 (1.00, 1.00)^∗^				
Perceived competence	0.85 (0.54, 1.16)^∗^				
Control	0.53 (0.20, 0.90)^∗^				
**Interactional empowerment**			0.87 (0.49, 1.48)^∗^		−0.00 (−0.44, 0.70)
Collective action		1.00 (1.00, 1.00)^∗^			
Interpersonal relations		0.91 (0.71, 1.17)^∗^			

*^∗^*p* < 0.05.*

**TABLE 5:** Indirect effects of media use on parental views and mediation.

**Dependent variables:**	**Intrapersonal empowerment**	**Interactional empowerment**	**Positive thoughts**	**Negative thoughts**	**Parental mediation**
**Independent variables:**					
**Social media use**					
Active use			0.09 (−0.03, 0.22)	0.00 (−0.07, 0.05)	0.14 (0.03, 0.26)^∗^
Passive use			0.14 (0.05, 0.25)^∗^		
**Control variables:**					
**Internet skills**	0.13 (0.05, 0.22)^∗^	0.15 (0.07, 0.26)^∗^	0.20 (0.08, 0.33)^∗^	0.00 (−0.18, 12)	0.35 (0.17, 0.53)^∗^

*^∗^*p* < 0.05.*

**FIGURE 2:**
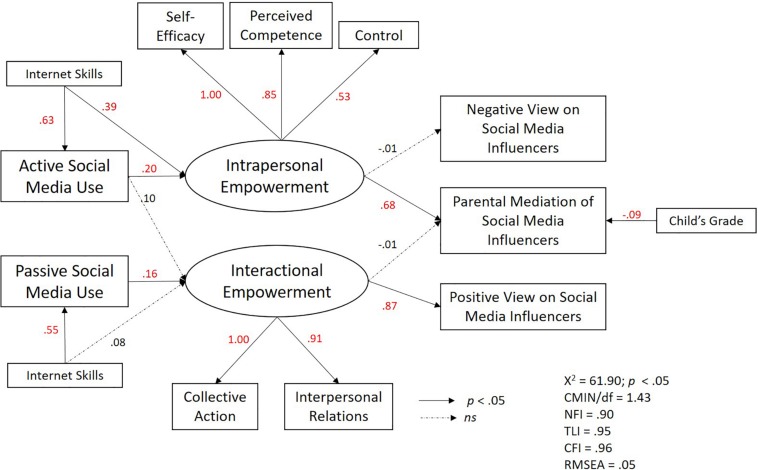
Final model.

For the final analyses, we tested and compared two models. Model 1, set up as per above expectations, predicted a direct relationship from parental views to parental mediation. However, Model 1 did not present a good fit to the data [χ^2^ (45) = 212.26, *p* < 0.001; CFI = 0.68; TLI = 0.53; RMSEA = 0.14, *p* < 0.001; 90% CI = (0.12, 0.16)]. In Model 2 (see model in Figure 2), the relationship between parental attitudes and parental mediation was removed (based on lack of correlation results between these variables) and parental mediation was predicted to be directly influenced by empowerment. This revision presented a better fit model [χ^2^ (43) = 61.59, *p* < 0.05; CFI = 0.96; TLI = 0.95; RMSEA = 0.05, *p* < 0.50; 90% CI = (0.02, 0.08); CMIN/DF = 1.43]. The model fit was considered good because the fit indices met the standard requirements of CFI > 0.95, TLI > 0.90, RMSEA < 0.05, and CMIN/DF was < 2.00 (Jöreskog and Sörbom, 1993; Hu and Bentler, 1999). The observed standardized factor loadings were significant and greater than 0.50 (Hair et al., 1998) for all of the indicators of the psychological empowerment variables.

### Direct Effects

The results of Model 2 suggest that active social media use had a significant effect on the latent variable, intrapersonal empowerment [0.20 (0.06, 0.30)], but not interactional empowerment, as expected, supporting H1. Although intrapersonal empowerment did not increase negative thoughts about social media influencers [−0.09 (−0.42, 0.23)], it increased parental mediation of the effect of social media influencers [0.68 (0.05, 1.22)], finding support for H2b but not H2a.

We find that passive social media use had a significant effect on the latent variable, interactional empowerment [0.16 (0.05, 0.27)], but not on intrapersonal empowerment, supporting H3. Interactional empowerment also increased positive thoughts about social media influencers [0.87 (0.49, 1.48)] but was not related to parental mediation [−0.00 (−0.44, 0.70)], supporting both H4a and H4b (see Table 4 for complete set of results).

When examining the control variables, parental Internet skills had a significant effect on passive use of social media [0.55 (0.35, 0.74)], active use of social media [0.63 (0.43, 0.83)], and latent variable, interactional empowerment [0.39 (0.19, 0.58)]. Additionally, a child’s grade level had a significant negative effect on parental mediation [0–0.09 (−0.18, −0.01)].

### Indirect Effects

The path model was also used to investigate the indirect effects of parents’ social media use on their views of social media influencers and level of parental mediation. Several indirect relationships emerged. As presented in Table 5, we find that active social media use, but not passive use, had a significant indirect effect on parental mediation *via* intrapersonal empowerment [0.14 (0.03, 0.26)], finding support for H5b. Furthermore, although active social media use did not result in more negative views of social media influencers, passive social media users had a significant indirect effect on positive views on social media influencers *via* interactional empowerment [0.14 (0.04, 0.25)], partially supporting H5a (see Table 5 for estimates).

## Discussion

Social media is becoming one of the dominant mediums for consumer–brand interactions (Dolan et al., 2016). Overall, the active use of social media by users is encouraged and viewed positively by firms. Many brands encourage their consumers to engage with them online for the purpose of increasing brand preference and sales (Pagani et al., 2011). Yet, we know little about how social media use shapes parenting. This study is one of the first to examine and provide an answer to that question. In fact, we demonstrate that a parent’s active use of social media trickles down and could lead to more mediation of their child’s encounters online with social media influencers. Moreover, we show that this can be explained by enhanced intrapersonal empowerment developed through active use of social media. Thus, through conducting this study, we not only respond to Frontiers in Psychology’s special issue call for a better understanding of the impact of social media influencers on our lives but also present positive findings related to the use of social media for both consumers and businesses. Some are more critical about the side effects of social media use and highlight the dark side of it (Scheinbaum, 2017), and we acknowledge that social media does present not only present positive but also certainly negative consequences from overuse, misuse, or irresponsible use. In this paper, however, we identify the benefits of social media use for preparing parents in their parenting practices for the modern age of the Internet.

Unlike traditional ads, Internet ads are of various types broadly lying in the continuum of explicit to implicit/native ads. The number and impact of social media influencers are growing by the day, and as researchers, we are only at the tip of the iceberg in understanding how the use of influencers in brand promotions is changing the marketing and consumption worlds (Lou and Yuan, 2019). Influencers, because of the nature of their trusted relationship with adolescents, are becoming a popular and powerful tool used by large and small brands alike. Children’s defense mechanisms are down when they are engaging with the posts of whom they follow and are much more easily influenced and swayed by a “friend” or even a stranger they “follow” online. This form of native advertising, in comparison to other more explicit forms of ads, requires a much-advanced level of cognition. The branded social media posts by influencers look and feel like regular, non-branded posts, thus making it difficult even for adults to differentiate commercial content from personal posts (Cornish, 2014; Folkvord et al., 2019; van Dam and van Reijmersdal, 2019). Researchers suggest that the nature of the ads may present an additional challenge to parents given parents’ limited recognition of these “newer” digital formats (Evans et al., 2013). For instance, Evans et al. (2013) found that parents struggled with understanding and mediating advergames. On the other hand, a banner ad that appears more like a traditional ad might be less challenging for parents to mediate (Evans et al., 2018). It appears that only when parents are equipped with that understanding can they help their adolescent. We demonstrate in this paper how social media use can play a role in providing parents that understanding and consequently impact their adolescent’s understanding of branded posts by social media influencers.

In sum, we find that when parents are actively engaged in the use of social media, they become better equipped with the social media marketing tactics, increasing their self-efficacy, competency, and control beliefs to mediate social media influencers and their impact on their adolescent. Specifically, active social media users showed higher intrapersonal empowerment, which, in turn, increases parental mediation of social media influencers’ impact, even after controlling for parental Internet skills and child’s grade. Passive use of social media, on the other hand, though increases interactional empowerment, tends to result in parents having more positive views of social media influencers. Perhaps this could be why it has no significant relation to parental mediation behaviors because parents do not see a need for it. The implications of these results are further discussed below.

Active users of social media are suggested to be innovators who might have an inherent interest in understanding how new forms of social media work and are clear about the benefits/disadvantages of using social media and how they could participate in social media (Pagani et al., 2011). Hence, it is likely that these parents may use their knowledge gained through active use of social media and apply appropriate mediation techniques. This finding may put some firms in a catch-22 situation. Firms, in general, would like users to create social media content, which increases a consumer’s engagement with the brand as it carries positive outcomes for the firm (Dolan et al., 2016). However, increased active engagement could also mean that once parents become more aware and knowledgeable of these tactics, they will be regulating and managing their adolescent’s social media use more. From a corporate social responsibility point of view, such outcomes, though unintentional, may allow firms to take credit for training and accustoming parents to become more skilled social media users. Parents themselves may also benefit from being actively engaged in the use of social media. Unlike how passive use of social media leads to increased envy of others, higher likelihood of depression, and decrease in well-being (Verduyn et al., 2015), active use of social media may increase personal well-being of the parent.

As we found in our analysis, active social media use is positively related to levels of Internet skills, but so does passive use of social media. This finding suggests that active use of social media captures capability beyond having Internet skills. In fact, active use of social media captures how parents put their Internet skills to use for the purpose of engagement, creation, and interaction. Thus, it is essential that researchers differentiate between active and passive use as our results demonstrate that different types of social media usage, unlike Internet skills, empower parents in different ways, resulting in varying levels of parental mediation.

Another critical finding in the study is the need to adopt nuance when we discuss empowerment. Psychological empowerment is made up of two distinct dimensions. Intrapersonal empowerment deals with a parent’s competency and belief in their abilities to mediate social media (Li, 2016). Interactional empowerment captures a parent’s reliance on others to handle their adolescent’s social media consumption (Fadda et al., 2017). The two appear to work in different and opposite ways, leading to different outcomes. Thus, researchers and policy makers should take note to consider the different dimensions of empowerment when conducting studies and/or implementing outcomes from such studies for the purpose of empowering individuals. Our finding also suggests that parenting information, whether on mediating social influencers or possibly other forms of mediation, needs to be provided in multiple ways considering the individual differences. A parent actively using social media might rely on regulator websites, news reports, mommy blogs, or sites such as Common Sense Media to educate themselves. In contrast, a parent passively using social media might rely on recommendations of school teachers or other parents in their network to act. It appears from our finding that such support is not currently available for parents passively using social media, explaining why these parents are not involved in mediation of social media influencers. Furthermore, if parents passively rely on the community and the norms for directing their parenting practices, then unless the community culture and norms point to the need and relevance of parental intervention, parents are unlikely to change their practices. In our study, we find that passive-use parents have a positive view of social media influencers, which may only shift to a more critical view if others around them feel the same way.

High levels of parental mediation could help mitigate the effects of Internet ads (American Academy of Pediatrics, 2016), which we believe applies to social media influencers as well. Specifically, we suggest that parents can be more involved in mediation by using a combination of co-surfing with their adolescent on social media, having open discussions about social media influencers, restricting the amount of time spent by the child on social media, and monitoring their adolescent’s use of social media. A combination of such interventions is likely to better prepare the child for marketing influences on the Internet even when a parent is not around.

### Limitations and Future Research

In this study, we gather responses only from mothers as they are usually the primary caregivers of adolescents and are also considered to be present on social media more than fathers. However, with the increasing participation of fathers in parenting as well nowadays, it is critical to consider their habits and views also. Future research should include fathers in their sample and possibly conduct multigroup analyses to see if fathers vs. mothers influence their adolescent’s social media use and responses differently. Our sample is also demographically slanted in terms of race/ethnicity and may not present a full picture across cultures and subcultures. While we do not find any significant impact of race/ethnicity, this could be the case because we did not have a balanced representation of the various groups in the sample. Future research could test the external validity of this study by considering this aspect as well.

The scope of this paper is to focus on parental attitudes and behaviors toward social media influencers, hence focusing on behaviors such as parental mediation. In the future, responses to social media influencers (or even brands) from the child could be captured and linked to the mediation methods that their parents use to determine the effectiveness of these methods. It is expected that adolescents whose parents mediate are less likely to be influenced by social media influencers’ claims and promotions. One can also expect these adolescents to also show higher levels of persuasive knowledge in regard of social media influencers and maybe even lower their purchase intention of the products promoted by these social media influencers.

Our data did not support a possible relationship expected between parental views and parental mediation. This could have happened for several reasons, including methodological limitations and possible other indirect mediating factors that would be at play. While traditional parental mediation models would suggest parental attitudes to predict parental mediation methods, it should be noted that known researchers studying Internet advertising have highlighted the fact that processing of native ads is very different from that of traditional ads (van Reijmersdal et al., 2012). Attitudes toward social media influencers might be more complex, in a sense that using a negative and positive attitude measure may not fully capture the nuances involved in predicting parental mediation behavior. Future research should consider other factors that could either better predict parental mediation or shall be considered along with attitudes toward social media influencers.

In this paper, we focused mostly on understanding the role of active (vs. passive) user’s interactions with social media. However, the way users interact with brands and social influencers on social media are likely to also depend on the content. Dolan et al. (2016) suggest that relational and entertainment content encourages active engagement, while informational and remunerative content will result in passive engagement. Future researchers could seek to understand how the nature of the content (entertainment vs. informational) may interact with the form of social media use, thus resulting in different perception and parental mediations.

Influencers, because of the nature of their trusted relationship with adolescents, can also act as role models who can inspire and change the lives of the users in a positive fashion (De Jans et al., 2018). Future research and practitioners should consider ways in which social media influencers could have a positive impact on adolescents in other aspects of life, such as forming good eating or learning habits, reaching beyond commercial purposes. This is also important because, as we see in this research, there is a bright side to the active use of social media. Finally, the results from this survey study are encouraging and further highlight the urgent need to conduct experiments by creating conditions of active or passive social media usage to causally test whether it increased/decreased intrapersonal and interactional empowerment.

## Conclusion

The research on parental mediation of Internet advertising is slowly but steadily growing. We believe that this study is one of the first to demonstrate that (a) parents vary in their attitudes toward social media influencers and level of mediation of social media influencers and (b) such variance can be explained by their own form of social media use. Overall, parents should be encouraged to engage with their adolescent actively on the topic of social media influencers. This is more likely to occur when parents themselves are more alert to the design and impact of social media influencers, which we find in our study is likely to occur when parents are more empowered through the active use of social media.

## Data Availability Statement

The raw data supporting the conclusions of this article will be made available by the authors, without undue reservation, to any qualified researcher.

## Ethics Statement

The studies involving human participants were reviewed and approved by the California State University Monterey Bay. The patients/participants provided their written informed consent to participate in this study.

## Author Contributions

M-HL and AV were involved in the planning, data collection, data analysis, and writing of this manuscript. RL was involved in planning and writing of this manuscript.

## Conflict of Interest

The authors declare that the research was conducted in the absence of any commercial or financial relationships that could be construed as a potential conflict of interest.
