# Supplementary material for: Toward an Understanding of Parental Views and Actions on Social Media Influencers Targeted at Adolescents: The Roles of Parents’ Social Media Use and Empowerment
Source: Front Psychol. 2019 Dec 6;10:2664. doi: 10.3389/fpsyg.2019.02664 (PMC6909972; doi:10.3389/fpsyg.2019.02664)
Supplement: Supplementary file 1 [file Table_1.DOCX]

## **Supplementary Material**

Social media influencer description that was provided to the parents as part of the questionnaire

*Who are social media influencers?*

*Social media influencers are people on social media featuring/reviewing a brand or product for a company. This could be by a celebrity, Youtuber, a friend or even family member that you are following. Companies reach out to social media users who have a large following to share a personalized message about the brand on their feed. In turn, these users get paid by the company or get the product for free.*

*This kind of advertising is typically found on Instagram, Facebook or Twitter. The influencers offer a personalized message on a brand that can create an emotional connection between the brand and the viewer. These messages often encourage children to purchase or support the brands.*
